# Evidence of slab tearing on an inherited Mesozoic rift transfer fault in the Betic Cordillera

**DOI:** 10.1038/s41598-025-13168-z

**Published:** 2025-08-07

**Authors:** Antonio Pedrera, Juan Díaz-Alvarado, Antonio Azor, Andrés Folguera, Luis González-Menéndez, Jesús García-Senz, Pedro Pablo Hernaiz-Huerta, Ana Ruiz-Constán, Carlos Marín-Lechado, Adrià Ramos, Berta López-Mir, Carmen Rodríguez, Roberto Jiménez-Borrego, Károly Hidas

**Affiliations:** 1 Instituto Geológico y Minero de España. Consejo Superior de Investigaciones Científicas (IGME-CSIC), Madrid, Spain; 2 King Juan Carlos University, Madrid, Spain; 3 University of Granada, Granada, Spain; 4 University of Buenos Aires, Buenos Aires, Argentina; 5 University of Alicante, Alicante, Spain; 6 Andalusian Earth Sciences Institute (IACT-CSIC), Granada, Spain

**Keywords:** Subduction zone, Rift inheritance, Slab tearing, Mantle upwelling, Betic Cordillera, Gibraltar Arc, Geochemistry, Geodynamics, Geophysics

## Abstract

The Western Mediterranean has undergone complex subduction and collision between the African and Iberian plates, influenced by slab segmentation and melt generation. Despite numerous studies aimed at understanding these connections, the style of subduction remains controversial. Utilizing a compilation of geophysical data and a new map of magmatic suites along the Western Betic Cordillera, along with geochemical and geochronological analyses, this paper presents a 3D reconstruction of a segmented subducting slab beneath the Gibraltar Arc, with a focus on the nature and timing of slab tearing and magmatism. Results suggest that magmatism was coeval with the retreating of subduction and slab tearing along the Antequera Fault Zone, a reactivated Mesozoic rift transfer fault. Slab tearing facilitated asthenospheric upwelling, triggering a localized thermal pulse in the upper plate during the Early Miocene. Zircon U–Pb geochronology witnesses this thermal event with the formation, emplacement, and crystallization of leucogranitic melts at low-pressure conditions, featuring both simple zircons (sometimes with inherited cores) and complex zircons (with rim dissolution and regrowth in host metamorphic units). Our findings demonstrate how inherited rift-related structures can drive slab tearing and asthenospheric upwelling, shaping the spatial and temporal patterns of magmatism and high-temperature metamorphism in complex subduction systems.

## Introduction

The geometry of subducted slabs provides fundamental constraints on the understanding of the tectonic, magmatic and metamorphic evolution of collisional orogens e.g.,^1–4^ particularly in complex 3D settings with strongly arcuate slabs containing both oceanic and continental subducted segments e.g.,^5–9^. Transfer faults inherited from previous rifting events, particularly former oceanic transform faults, are considered key elements during orogenesis since they can be reactivated as vertical tear faults during subduction and promote segmentation during collision e.g.,^10,11^. Vertical slab tearing controls the subduction retreat pattern and creates steep channels that allow asthenospheric-derived magmas to reach shallow crustal levels^12,13^These asthenospheric thermal pulses can promote high-temperature metamorphic processes including crustal anatexis in the upper plate e.g.,^14–17^.

**Fig. 1:**
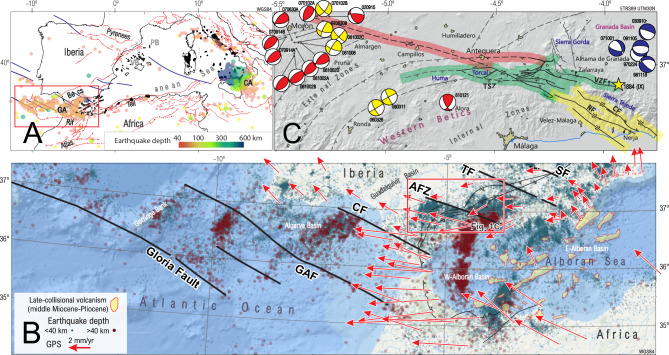
(**A**). Tectonic map of the Western Mediterranean highlighting seismicity distribution. Note the contrast in earthquake depth between the subduction beneath the Calabrian Arc (CA), where seismicity extends to greater depths, and the Gibraltar Arc (GA), where it reaches intermediate-depth. Miocene to Quaternary volcanism in the Western Mediterranean is highlighted in black (redrawn from ^74,108,109^). VB, PB are the Valencia and Provençal Basin. (**B**) Simplified map showing the relationship between transform and transfer faults extending from the Gloria Fault, the Algarve Basin, and inverted in the Betic Cordillera with the seismicity distribution (GAF: Gorringe-Algarve Fault; CF: Cádiz Fault; AF: Antequera Fault Zone: TF: Tíscar Fault; SF: Socovos Fault) (https://www.ign.es/web/ign/portal/sis-catalogo-terremotos. 2024.). Modified from Pedrera et al., 2020. Post to late-collisional magmatism is highlighted in yellow (redrawn from Duggen et al.,^74^). GPS velocity field^49^^,^^50^ shows a uniform 2–4 mm/yr westward movement south of the Antequera Fault Zone. (C) Map of the Antequera Fault Zone, highlighting its division into northern, central, and southern segments, shaded in red, green, and yellow, respectively. Solutions for earthquake focal mechanisms corresponds to^51,110–113^. Maps generated with QGis Desktop 3.40.1 (https://qgis.org/), Move Suite 2018.1 (https://www.mve.com/) and Corel Draw Graphics Suite X8 (https://www.coreldraw.com/).

The Mediterranean region, surrounded by orogenic arcs and associated back-arc basins e.g.,^18,19^, provides an exceptional setting to further investigate the relation of slab subduction, tectonic inheritance and asthenosphere upwelling. One of the tightest oroclines in the region is the Gibraltar Arc in the Western Mediterranean, which is associated with the formation of the Betic-Rif Cordillera and involved the subduction of Mesozoic oceanic crust and slab retreat beneath the Alboran Basin (Fig. 1A). The proposed subduction dynamics are primarily based on seismic tomography models that reveal a high-velocity P-wave anomaly in the upper mantle beneath the Gibraltar Arc e.g.,^20–23^. Most models consistently identify this anomaly as a subducted oceanic lithospheric slab; however, there is significant variation in its interpreted lateral extent and depth. While some studies confine the anomaly to the Western Betics and the Rif e.g.,^23^, others extend it further east beneath the Central and Eastern Betics e.g.,^20–22^. Depth estimates also vary widely; Palomeras et al.^23^ image the anomaly down to ~ 125 km, whereas other models suggest it continues to depths of up to 600 km^20–22^. Interestingly, intermediate-depth seismicity is restricted to the western Betic Cordillera, west and south of the Antequera Fault Zone, and reaches depths of only ~ 130 km^23^ (Fig. 1B). Furthermore, paleogeographic reconstructions and restored crustal-scale cross-sections constrain the amount of shortening more precisely, suggesting that the actual slab length is considerably shorter than previously proposed e.g.,^24,25^. These apparent contradictions have given rise to contrasting geodynamic models: a group invoking large-scale west-directed slab rollback with E-W to ENE-WSW slab tearing^18,20,26^ another group proposing a continuous southwest-dipping oceanic slab beneath the Betic Cordillera^27,28^ and a third proposal suggesting a southeast-dipping oceanic slab, limited to the west of the Antequera Fault Zone and transitioning to continental subduction beneath the Central Betics^24^. This apparent mismatch highlights the need to better constrain the position and kinematics of slab tear faults, which remain poorly understood and are critical for resolving the geodynamic evolution of the region.

This study presents a 3D reconstruction of the subducting slab beneath the Gibraltar Arc down to a depth of 130 km, based on a compilation of geophysical data, including borehole data, seismic reflection profiles, receiver function images, and seismicity distribution. The model allows us to characterize major slab tear faults in the Betic Cordillera, identified from abrupt seismicity terminations and discontinuities in receiver function images. Newly produced geological maps further define the structure of the Antequera Fault Zone and document the distribution of magmatic suites in the Western Betics. Combined with geochemical and geochronological analyses (published data and new samples), these results constrain the timing and nature of magmatic processes. Together, the findings link high-temperature metamorphism and magmatic activity to slab tearing dynamics, offering key insights into the geodynamic evolution of the Western Mediterranean.

## Geological setting

The Betic Cordillera, together with the Rif Mountains, forms the westernmost tectonic arc of the Mediterranean system (Fig. 1A). It underwent Mesozoic rifting and limited oceanic spreading between the African and the Eurasian plates, followed by subduction and collision of the conjugate rift margins [e.g.^24,28–30^]. First-order WNW-ESE trending inherited rift transfer faults (Gloria, Gorringe-Algarve; Cádiz, Antequera, Tíscar, Socovos; Fig. 1B) have been identified within the orogen [e.g.^24,31^; Fig. 1B]. The most prominent is the Antequera Transfer Fault (Fig. 1C) that separated a relatively wide domain of hyperextended continental lithosphere to the east from a narrow domain of oceanic crust to the west^24^.

**Fig. 2:**
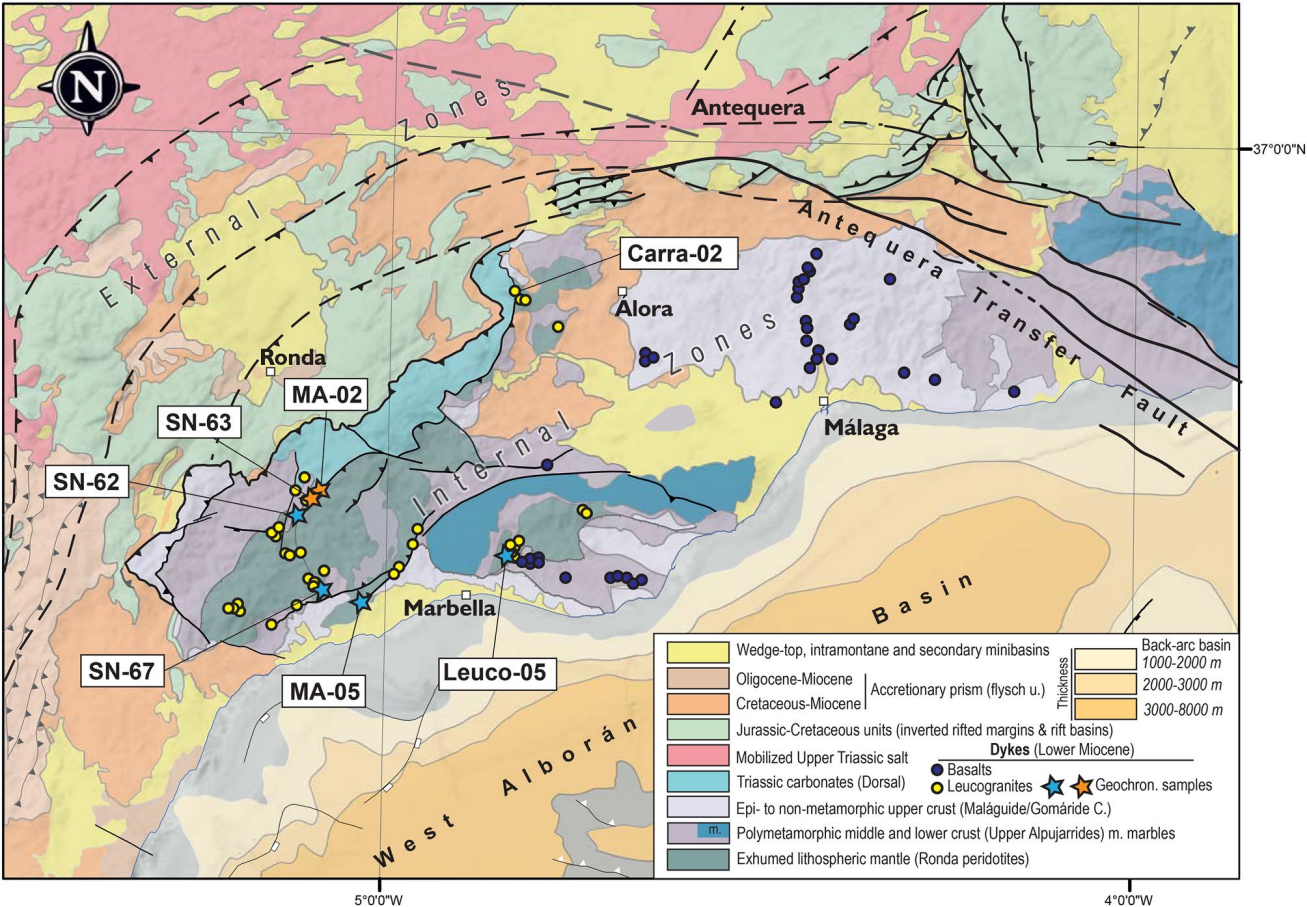
Geological map with the location of the Antequera transfer fault and the location of magmatic suites. The location of the samples used in the geochronological study are marked. Note the spatial relationship between the location of the main dykes and small intrusive bodies of leucogranites and the major structural lineaments reactivated during the Miocene. Map generated with QGis Desktop 3.40.1 (https://qgis.org/), Move Suite 2017.1 (https://www.mve.com/) and Corel Draw Graphics Suite X8 (https://www.coreldraw.com/).

The External Zone of the Betic Cordillera is composed of a syn-rift Mesozoic sedimentary sequence, including a thick succession of Upper Triassic evaporites (Fig. 2) [e.g.^32^]. The Internal Zone include metamorphic units formed during the pre-collisional and syn-orogenic tectonic evolution. The orogen includes Cretaceous to Miocene pelagic rocks of the Flysch Units, interpreted to overlay oceanic and/or attenuated continental crust west of the Antequera fault^33^ (Figs. 1B and 2). In the rest of the orogen, these units unconformably overlay the Internal Zones and form an accretionary wedge between the Internal and External Zones. The Guadalquivir foreland basin occupies the frontal part of the orogen, while the western Alboran back-arc basin is in the hinterland (Figs. 1 and 2).

In the Western Betics, the Internal Zone comprises, from top to bottom: the Malaguide units, composed of non-metamorphic upper crustal rocks; and the Alpujarride units, composed of mid-lower crustal metamorphic rocks and peridotites from the lithospheric mantle e.g.^34^ (Fig. 2). The western Alpujarride units include high-pressure metamorphic relicts^35,36^, overprinted by S-L fabrics indicative of decompression and heating. This low-pressure heating process is linked to strong extensional lithospheric attenuation, dated to around 280 Ma (Early Permian) in the crustal units above the Ronda peridotites e.g.^34,37^.

Superposed to these previous processes, Early Miocene radiometric ages linked to a high-grade metamorphism event are recorded by several thermochronometers with different closure temperatures in most of the western Alpujarride units^38–43^. Late Oligocene (?)—early Early Miocene synorogenic magmatism is limited to the western Betics, where granitic and basaltic dykes are scarce e.g.,^44–46^. Calc-alkaline magmatism is absent in the western Betics and is instead confined to an ENE-WSW trending band extending across the Alboran Basin, from the eastern Betics to the eastern Rif, during a post-collisional phase spanning the middle Miocene to the Pliocene e.g.,^47^(Figs. 1A and B).

## Results

### The Antequera Fault Zone

The Antequera Fault Zone is a fundamental structural element of the Betic Cordillera. It significantly influences the distribution of Mesozoic domains, the partitioning of deformation during subduction and collision, and the current kinematics and seismicity^24^. From a regional perspective, it is part of a system of parallel transform and transfer faults extending from the Gloria Fault, through the Algarve Basin, to the former Betic realm (Fig. 1B). These accommodated extensional strain variations during the Late Jurassic-Cretaceous. During the subsequent Alpine orogeny, the Antequera Fault Zone played a crucial role in differential shortening distribution, marking the northern limit of the western Gibraltar Arc^48^.

This structural influence persists today, as reflected in the regional GPS velocity field. South of the Antequera Fault Zone, in the western Betics, GPS measurements indicate a uniform W-NW movement of 2–4 mm/yr. In contrast, north of the fault, velocities decrease significantly to 0.5–1 mm/yr and shift to a dominant W-SW direction^49,50^ (Fig. 1B). The Antequera Fault Zone branches out into several subsidiary faults affecting basement rocks, following a general N110°E strike. It records a general dextral strike-slip deformation pattern with both transpressional and transtensional domains. Three main fault segments (northern, central and southern) can be identified (Fig. 1C).

The northern segment (N105-110°E) extends 70 km between Antequera and Morón (Fig. 1C). It forms a boundary for crustal seismicity, which is partitioned and largely confined to the southern block of the fault (Fig. 1B). In the Morón area, focal mechanism solutions indicate that the fault acts as a dextral shear zone, connected with ENE-WSW reverse faults toward the front of the orogen^51,52^. This fault segment lacks a clear surface expression due to thick Upper Triassic evaporites, which decouple deformation between the basement and cover and facilitate salt mobilisation into higher stratigraphic levels (Fig. 2). As a result, its presence at the surface is only subtly expressed by the alignment of secondary minibasins and the occurrence of limestone and dolostone stringers, commonly exposed within mobilised evaporites and clays (Fig. 2

The central segment (N90-100°E) forms a prominent structural feature, which is exposed for about 50 km between the Torcal Range and Zafarraya (Fig. 1C). It consists of multiple interconnected and anastomosing dextral transpressive faults [e.g.,^53^] that deform both the basement and the overlying Mesozoic succession. The interaction between WNW-ESE dextral strike-slip faults and E-W thrusts results in a characteristic rhomboidal pattern of fault-bounded slivers (Figs. 1C and 2). These bound isolated ranges, generally composed of Jurassic and Cretaceous carbonate sequences. Stratigraphic contrasts between these ranges reflect the activity of the fault system during the Mesozoic. In the southwest, the Lower Cretaceous is absent or reduced to a condensed interval over an erosional surface, marked by Neptunian dykes infill and condensed facies. To the northeast, it forms a continuous marly succession^54^.

To the SE of this fault system, around Colmenar, another network of transpressive interconnected faults cut across the Maláguide units, promoting the deposition of Eocene-Burdigalian syn-orogenic sediments (Figs. 1C and 2). These faults extend along the southern boundary of Sierra Gorda and are linked to the Ventas de Zafarraya Fault, with normal to normal-dextral transtensional kinematics e.g.^55^.

The southern segment (N100°E) extends for about 30 km from the southwestern margins of the Tejeda-Almijara and Competa Sierras to the coastline (Fig. 1C). It consists of an array of sub-parallel faults with dextral, dextral-normal, and normal kinematics e.g.^56,57^.

### Geometry of the subducting slab interface and vertical tear faults

**Fig. 3:**
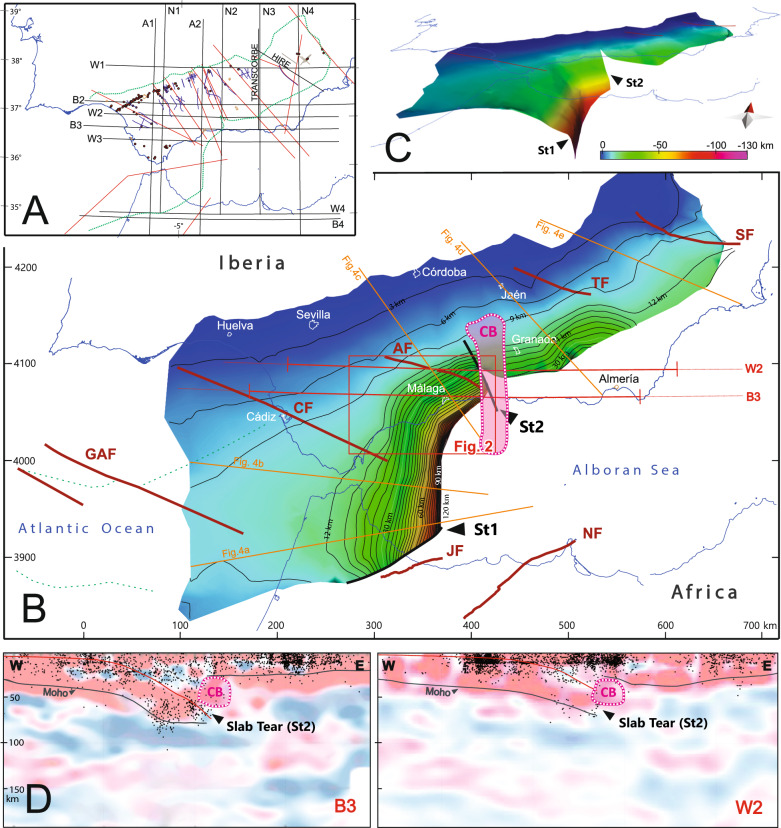
(**A**) Map showing the location of cross-sections^24^ and published P-to-S converted receiver function migrated images used for constructing the 3D model^57–61^. (**B**) 3D representation of the top of the Iberian subduction slab, highlighting slab tear locations, Slab Tear 1 (ST1) y Slab Tear 2 (ST2). The conductive body (CB)^62^, location of map from Fig. 2, seismicity cross-sections from Fig. 4, and P-to-S converted receiver function migrated images from Fig. 3D are indicated (GAF: Gorringe-Algarve Fault; CF: Cádiz Fault; AF: Antequera Fault Zone: TF: Tíscar Fault; SF: Socovos Fault; JF: Jebha Fault; NF: Nekor fault). (**C**) Oblique view of the subducted slab. (**D**) Re-interpreted P-to-S converted receiver function migrated images (W2 and B3) [^59^, with permission of Elsevier], perpendicular to the Antequera Fault Zone, showing projected seismicity (black dots) and the intersection with CB. Maps, section and 3D view generated with QGis Desktop 3.40.1 (https://qgis.org/), Move Suite 2017.1 (https://www.mve.com/) and Corel Draw Graphics Suite X8 (https://www.coreldraw.com/).

The 3D geometry of the subduction slab interface was reconstructed using seismic reflection profiles, regional cross-sections, seismicity distribution, and published P-to-S receiver function migrated images^58–61^ (Fig. 3A). This surface represents the top of the basement of the Iberian Plate and the top of the attached oceanic crust. The flexural curvature of the subducted slab is disrupted by two vertical tear faults (ST1 and ST2; Fig. 3B,C). The southern one (ST1) is located beneath the Rif Cordillera and strikes WSW-ENE, while the northern one (ST2) is located beneath the Betic Cordillera and strikes NW–SE, forming 25° with the WNW-ESE Antequera Fault Zone in the upper plate. The northern tear fault (ST2) is characterized by: (i) a sharp step at the Moho and the top of the subducting interface; (ii) significant lateral variations in top slab dip and depth at either fault side; and (iii) an abrupt easterly-directed vanishing of intermediate-depth hypocentres (Fig. 3D). The northern vertical tear fault (ST2) coincides with a conductive body (CB), imaged by magnetotelluric surveys at depths between 30 and 50 km^62^.

**Fig. 4:**
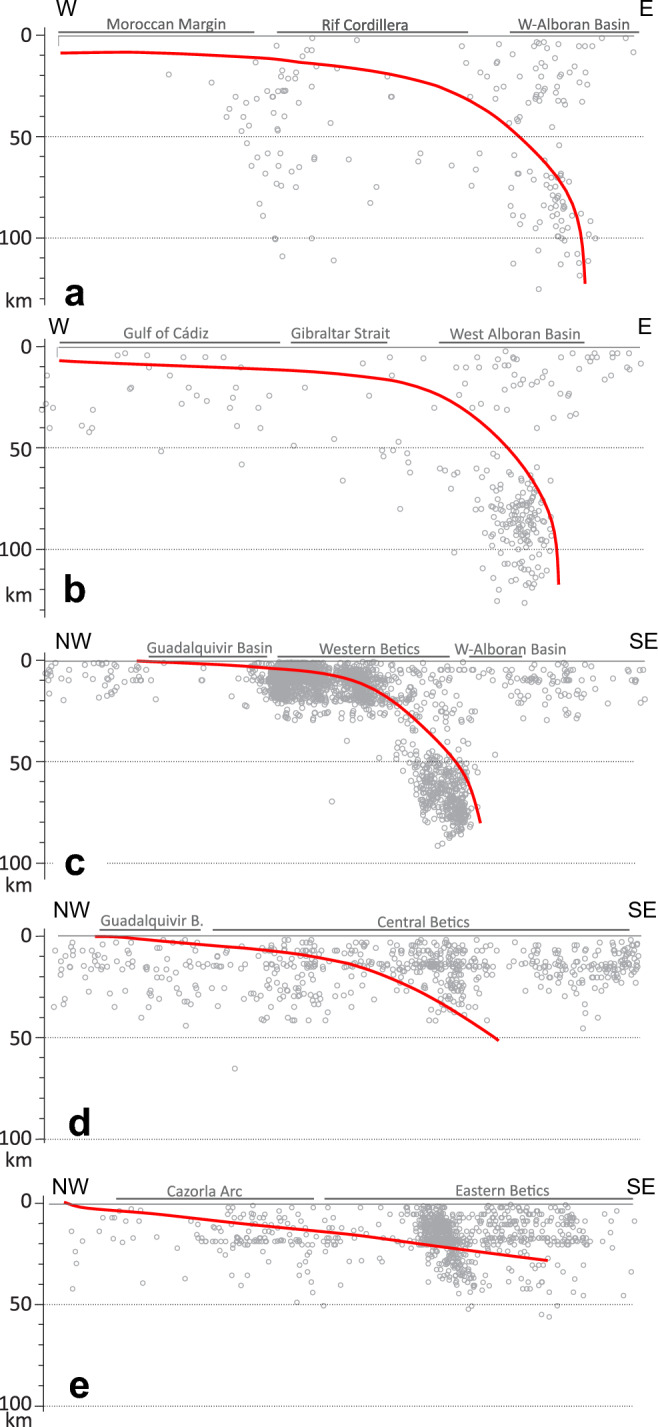
Cross-sections showing the geometry of the subduction slab interface (red line) and its relationship with the seismicity distribution. Location is marked in Fig. 3. The figure highlights variations in slab morphology on either side of the Antequera Fault Zone. Seismicity based on the Instituto Geográfico Nacional IGN^97^. Figure generated with Move Suite 2017.1 (https://www.petex.com/pe-geology/move-suite/) and Corel Draw Graphics Suite X8 (https://www.coreldraw.com/).

The subducting slab shifts from N10°E beneath the western Alboran Basin to N70°E beneath the western Betics (Fig. 3C). Variations along-strike are evident in both dip angle and subduction depth (Fig. 4). The slab falls to depths of about 90 km near the Antequera Fault, reaching 120 km depths further south. In a cross-section along the Strait of Gibraltar, the dip progressively steepens from ~ 7°-12° at 50 km-depth to nearly vertical (80°–90°) at 120 km-depth beneath the western Alboran Basin (Fig. 4B). Similarly, below the western Betic Cordillera, the dip transitions from ~ 7°-12° beneath the Guadalquivir foreland basin to approximately 80° below the western Alboran Basin, with the steepest portions occurring between 50–120 km depth (Fig. 4C). East of the Antequera Fault, in the Central Betics, the top of the Iberian basement beneath the Guadalquivir foreland basin dips gently (5°-6°), to reach a moderate inclination of ~ 30° beneath the Internal Zone (Fig. 4B,E).

### Magmatism in the Western Betics

The Internal Zone in the Western Betics hosts a network of steeply-dipping dykes of dioritic and leucogranitic composition (Fig. 2). Mafic dykes, referred to as the Málaga dykes [see^59^], are restricted to the south of the Antequera Fault Zone (Fig. 2). These dykes have an average thickness of 3–4 m and fairly constant orientations (E-W strikes and sub-vertical dips). Petrographically, they are described as fine-grained porphyritic to subophitic Pl-Hbl diorites, with minor Cpx, Qtz and rare Bt, with variable degrees of secondary alteration^63^ (abbreviations by^64^).

Leucogranites are more frequently exposed west of Álora (Fig. 2), forming sub-vertical dykes that range from a few cm to 10 m in thickness (0.5–1 m-thick on average). These volumetrically scarce felsic rocks typically intrude peridotites along serpentinized faults, but also cut through crustal metamorphic rocks along prominent brittle to ductile–brittle fault zones located west of the Antequera Fault Zone (Fig. 2). Thus, leucogranite dykes are situated within and close to regional-scale high-angle faults, usually paralleling these brittle structures. This suggests that pre-existing crustal weaknesses played a key role in facilitating magma ascent^34^. The present-day contacts of these dykes with the host rock are typically high-angle reverse to reverse-lateral faults, associated with significant serpentinization when crosscutting peridotites.

#### Petrology and geochemistry of the felsic melts

Cordierite and tourmaline are two of the few ferromagnesian phases found in the leucogranites. From a compositional perspective, these rocks have low Mg (MgO < 1 wt%) and variable Fe contents (1.5–3.4 wt% FeOt), including some samples with moderate B parameters (i.e., FeO + MgO + MnO) ranging from 30 to 80, thus differing from the composition of felsic peraluminous melts. The presence of Crd also explains the high peraluminosity of some of these leucogranites, which have an Aluminium Saturation Index (ASI) ranging between 1.12 and 1.46). For example, the mesocratic granite Leuco-05 has an ASI of 1.46. In the case of the leucosome sample SN-63, Crd is abundant in both the melanosome bands and the metatexite in which the leucosome is rooted. Characteristic of low-pressure migmatites, Crd is typical of peraluminous S-type granites formed during anatexis of pelitic metasediments by incongruent melting reactions (Bt breakdown) e.g.,^65,66^. The presence of peritectic Crd in peraluminous leucogranites has been the subject of numerous experimental, textural, and mineralogical studies, which unanimously point out to a pressure limit on the partial melting process (and the source area of the leucogranites) to a maximum of about 0.4–0.6 GPa e.g.,^67–70^.

The high silica (> 70 wt%), alkaline (Total alkalis > 6.5 wt%), and peraluminous (ASI > 1.2) leucogranites emplaced as tabular bodies south of the Antequera Fault Zone are clearly related to a metapelitic crustal protolith e.g.,^68,69^. Even though the Lower Miocene contractional event was preceded by a prolonged subduction period, there is no evidence for the presence of calc-alkaline magmatic rocks such as granodiorites and tonalites.

#### Geochemistry of the mafic melts

**Fig. 5:**
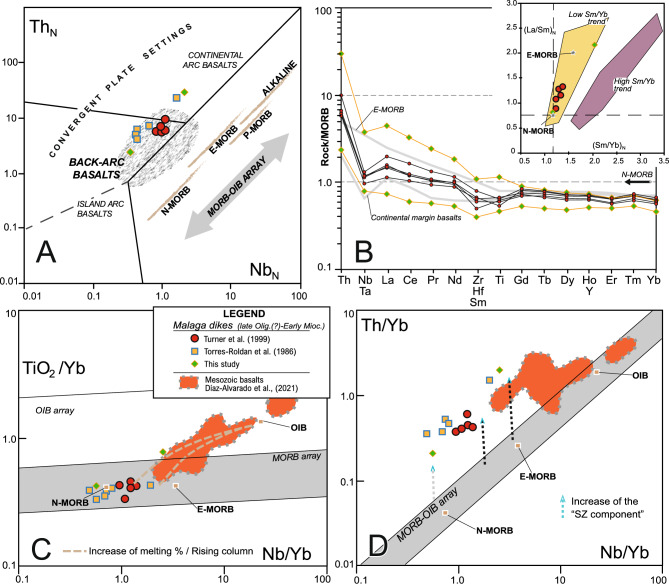
(**A**) Tectonic discrimination, MORB-normalized Th vs. Nb diagram after Saccani^102^. (**B**) Normal Mid-Ocean Ridge Basalt (N-MORB) normalized trace element patterns of the studied samples (Supplementary Material 1). Standard compositions of MORB, Enriched Mid-Ocean Ridge Basalt (E-MORB), and Ocean Island Basalt (OIB)^114^, and continental margin basalts^115^ are included for comparison. Inset shows the relation between normalized La/Sm and Sm/Yb ratios. Stars represent N- and E-MORB averaged compositions from Gale et al.^116^. (**C**) and (**D**) TiO2/Yb vs. Nb/Yb and Th/Yb vs. Nb/Yb ^117^. Mesozoic basalts from the Subbetic Basin (External Zone, Central Betics) are plotted for comparison [see ^29^]. Figure generated with Corel Draw Graphics Suite X8 (https://www.coreldraw.com/).

The Málaga dykes constitute a subalkaline to calc-alkaline series, comprising basalts and basaltic andesites, as inferred from their low Ti and Nb values^71^ and other features derived from major and trace elements (Fig. 5) e.g.,^72,73^ (Supplementary Material 1 and 2). They show high ^143^Nd/^144^Nd and low ^87^Sr/^86^Sr isotopic ratios^43^. Regarding multi-element diagrams, the Málaga dykes exhibit enrichment relative to Mid-Ocean Ridge Basalts (MORB) in Th and Light Rare Earth Elements (LREE), accompanied by a significant Nb–Ta anomaly akin to that observed in continental margin basalts (Fig. 5). The Mid Rare Earth Elements (MREE) and Heavy Rare Earth Elements (HREE) display a flat pattern, slightly depleted as compared to the MORB. These observations are further supported by the low Sm/Yb ratios and the more variable La/Sm ratios in the studied samples (Fig. 5), which consistently fall into the field of hypabyssal basaltic rocks from convergent plate settings with calc-alkaline affinity. Remarkably, most of the investigated samples align within the region defined by basalts related to continental back-arc or suprasubduction settings.

#### Age of mafic and felsic magmatism

Mafic magmatism in the Internal Zone of the Western Betics is recorded by a sub-vertical dyke complex (i.e., the Málaga basaltic dyke), suggesting a short-lived injection process. This has been constrained using published K/Ar and ^4^^0^Ar/^3^^9^Ar radiometric data, although these are limited by the scarcity of suitable minerals in mafic rocks. Reported radiometric ages include 22–23 Ma (K/Ar;^63^) and 30.2 ± 0.9 Ma (^4^^0^Ar/^39^Ar;^47,74^). Similarly, concordant U–Pb zircon ages are also restricted by the low zircon abundance, the presence of inherited or xenocrystic grains, and issues such as lead loss or metamictization (which can lead to discordant results). Despite these challenges, the few reported zircon ages range between 20.9 to 33.1 ± 0.7 Ma^75^, which fall within a similar age range as the K/Ar and the ^4^⁰Ar/^3^⁹Ar radiometric ages.

**Fig. 6:**
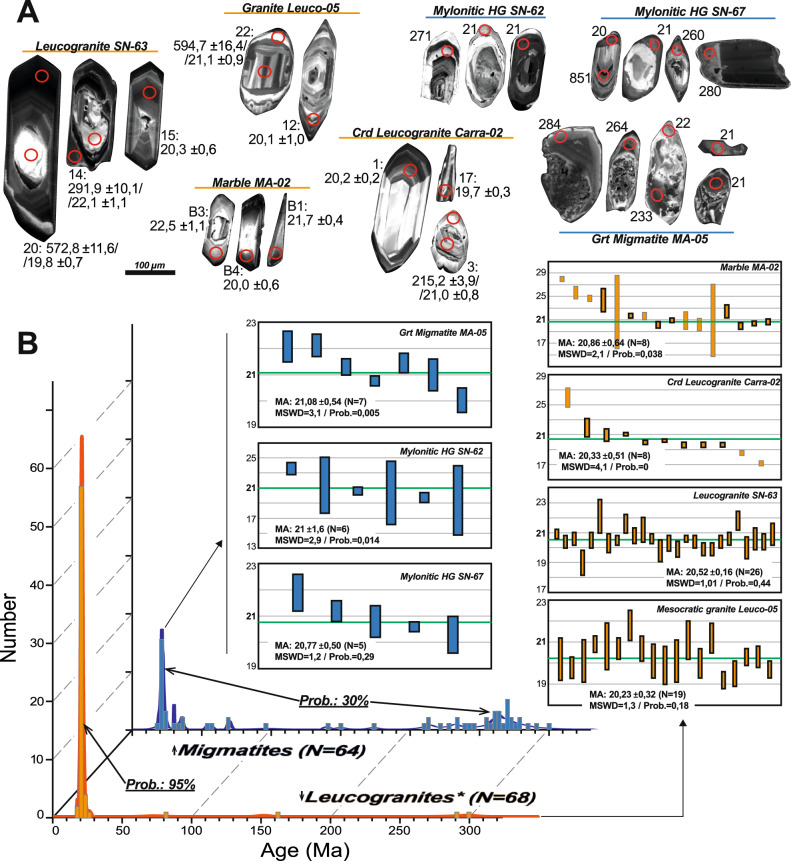
**A**) Representative zircons from the leucogranite, marble, and migmatite samples studied in this work. The location, description of samples and zircon populations and geochronological results and are provided in Supplementary Material 1, 3, and 4. **B**) Histogram and probability density plot of leucogranites and the marble sample MA-02 (orange), and migmatites (Grt-metatexites and nebulites or heterogeneous granites) (blue). The probabilities of the different age groups were obtained from the linearized probability plot. Boxes show the weighted average (MA: mean ages) of the seven samples. Error boxes heights are 1σ. Figure generated with Corel Draw Graphics Suite X8 (https://www.coreldraw.com/).

Felsic magmatism in the Internal Zone of the Western Betics is linked to the formation, emplacement, and crystallization of leucogranitic melts, the age of which has been constrained by U–Pb zircon dating on new samples (Fig. 2). Seven samples, four leucogranites and three migmatites, were dated using U–Pb zircon geochronology with the Sensitive High-Resolution Ion MicroProbe (SHRIMP), yielding ages for both simple zircon grains and overgrowths on inherited cores. The ^206^Pb/^238^U ages with low discordance fall within 22 and 19 Ma, resulting in mean ages between 20.2 and 20.5 Ma (Fig. 6) (Supplementary Material 1, 3 and 4). This uniform age distribution contrasts with the age distribution in granites s.l. from larger igneous bodies in subduction or post-collisional contexts, even those of smaller dimensions, which usually have Gaussian distributions with a large age range. This distribution is related to their sequential emplacement, including antecrysts and autocrysts e.g.,^76^. Additionally, the larger the volume housed in the intrusive bodies, the greater the range of estimated ages for their construction^77^.

The dissolution and growth processes of zircon rims over inherited and previous magmatic/metamorphic zircons have also been studied in some metamorphic units of the study area (Fig. 2), which are also restricted to the main reverse-oblique high-angle faults (Fig. 2). The obtained ages (samples MA-05, SN-62, and 67) show greater variation than the ages obtained for the leucogranites, possibly due to the age resetting of Permian zircons during the Miocene thermal pulse. The metamictic alteration of these zircons is evident in sample MA-05. Nevertheless, the concordant or low discordant results form a mean age group around 21 Ma, which coincides within error with the age of the leucogranites.

## Discussion

**Fig. 7:**
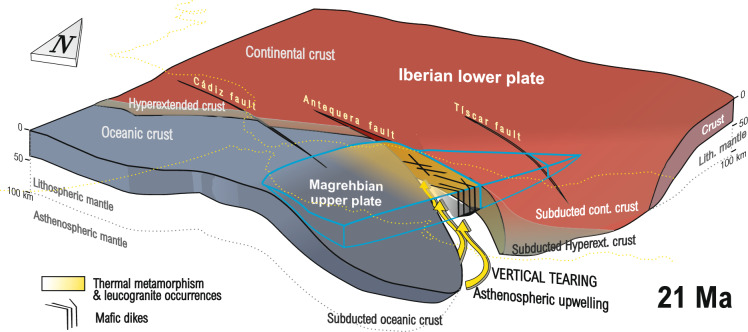
3D reconstruction of the Gibraltar Arc subduction at 21 Ma, illustrating the localization of slab tearing and asthenospheric upwelling along the inherited Antequera Transfer Fault. This process influenced the spatial and temporal distribution of magmatism and high-temperature metamorphism in the upper plate. 3D view generated with QGis Desktop 3.40.1 (https://qgis.org/), Move Suite 2017.1 (https://www.mve.com/) and Corel Draw Graphics Suite X8 (https://www.coreldraw.com/).

The geographical distribution, geochemistry, and age of magmatism provide critical constraints on the complex 3D geometry and dynamic evolution of the subducting Betic slab. Spatial and temporal relationships between mafic/felsic dykes, and thermal reactivation processes are recorded by zircon recrystallization and age resetting in host metamorphic rocks. Thus, the mafic Málaga dykes, located above and immediately south of a vertical lithospheric tear fault, roughly coinciding with the Antequera transfer fault in the upper plate, provide direct evidence of a short-lived mantle flow in the region supported by their narrow emplacement age range. Their geochemical signatures are characterized by the interplay between calc-alkaline and tholeiitic basaltic series, pronounced Nb–Ta anomalies, and Th enrichment, supporting a subduction-related origin (Fig. 5B,D). Furthermore, the absence of HREE fractionation and low Ti content reveals a slightly enriched mantle source and shallow decompression melting (Fig. 5B,C). These geochemical features are indicative of a suprasubduction origin (Fig. 5A). In the Western Betics, this setting corresponds to the retreat of the southeast-directed subducting oceanic lithosphere beneath the Maghrebian margin (Fig. 7). Thus, this paper proposes that slab rollback induced a toroidal mantle flow, in which material originally beneath the slab migrated around its lateral edge into the mantle wedge (Fig. 7). This interpretation aligns with multiple models in similar geodynamic settings such as the Pacific, South America, the North America, and the central and eastern Mediterranean subduction systems e.g.,^78–82^. Asthenospheric flow, triggered by slab rollback and vertical tearing, emerges as the primary driver of metamorphic and melting processes during the subduction-to-collision transition in the Western Betics, influencing the lithosphere thermal state of at around 21 Ma (Fig. 7). Heating associated with asthenospheric upwelling reached mid-crustal levels, resulting in low pressure (< 0.4–0.6 GPa) melting of metapelites, which gave way to leucogranitic magmatism. Basaltic dykes predominantly intruded upper-crustal levels, whereas leucogranites were emplaced at mid-upper crustal depths. These differences are attributed to the higher viscosity of leucogranitic magmas as compared to basaltic ones e.g.^83^. The described field relationships, together with geochemical and geochronological data of the leucogranites, reveal that the thermal pulse was brief, lasting approximately 1 Ma. However, there is no evidence for a widespread Lower Miocene anatectic domain.

Partial melting and the formation of new zircon crystals and rims were confined to major high-angle fault zones. This localized temperature rise resulted from fluid circulation along high-angle faults, as proven by elevated boron concentrations in leucogranites^84^ and intense serpentinization of the host peridotites e.g.^85^. Furthermore, this thermal metamorphism explains the observed metamictization and isotopic disequilibrium in Permian zircons. Therefore, the convergence of isotopic ages with varying closure temperatures, together with the lack of correlation between age and metamorphic gradient, supports a significant resetting event in the Early Miocene, occurring at different crustal levels^46^. The presence of a conductive body aligned with the vertical slab tear fault suggests that the thermal anomaly persists, indicating that the lithosphere in this sector has not yet fully equilibrated. Elevated temperatures have been maintained at depth long after the Lower Miocene magmatic event, possibly due to slow heat dissipation and/or residual mantle upwelling.

**Fig. 8:**
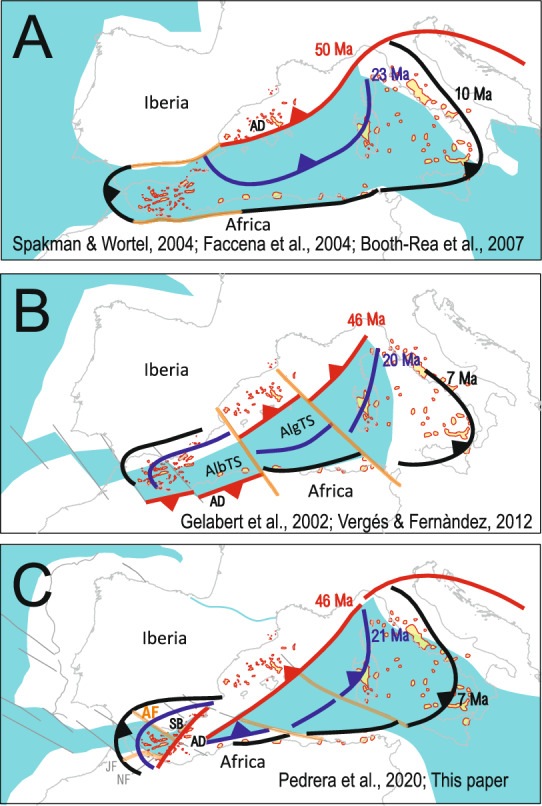
Existing geodynamic models for the Western Mediterranean, highlighting the role of tear faults (in orange) in the regional tectonic evolution (AlbTS: Alboran-Tethys slab; AlgTS: Algerian-Tethys slabs; SB: Subbetic Basin; AD: pre-orogenic location of the Alboran Domain in different models; AF: Antequera fault; JF: Jebha Fault; NF: Nekor fault). In model C, the Nevado-Filábride Complex is interpreted, in its pre-orogenic position, as a hyperextended continental domain underlying the Subbetic Basin—a failed rift branch connected to the Atlantic. Miocene to Quaternary volcanism in the Western Mediterranean is highlighted (redrawn from^74^^,^^108,109^). Figure generated with Corel Draw Graphics Suite X8 (https://www.coreldraw.com/).

These findings contribute to ongoing debates about the timing and mechanisms of subduction retreat, slab segmentation, and the role of inherited structures in shaping the Betic-Rif Cordillera within the Western Mediterranean. Three primary geodynamic models have been proposed differing in the paleogeographic reconstructions before orogenesis and the subsequent geometry and evolution of subduction zones (Fig. 8). One group of models hypothesizes a large-scale westerly-directed rollback of a long north- to northwest-dipping subduction zone, initially extending along the southern Balearic block boundary, with subsequent E-W to ENE-WSW slab tearing and crustal delamination close to the slab edges (Fig. 8A) e.g.,^18,20,26,59^. This proposition suggests the westward displacement of the Betic-Rif Internal Zone, positioned atop the retreating slab, with the migration accommodated by the development of ENE-WSW to E-W trending tears in both the Betic and Rif cordilleras e.g.;^20^. A second group of models postulates lateral change on subduction polarity, with a southwest-dipping slab beneath the Maghrebian margins and a northwest-dipping slab beneath the southern Balearic block (Fig. 8B) e.g.,^27,28^. The shift in subduction polarity is believed to take place along a NW–SE transform zone separating the Alboran-Tethys and the Algerian-Tethys slabs (Fig. 8B). The third group suggests a southeast-dipping slab limited to an oceanic domain west of the Antequera Fault Zone, transitioning into continental subduction east of the fault, beneath the Central Betics (Fig. 8C)^24^. This geodynamic framework provides a coherent explanation for both the geometry of the slab top and tear faults imaged in this study, as well as for a broad array of independent geological, topographic, magmatic, and geophysical observations. For instance, our proposed slab geometry shows strong spatial correspondence with the high-velocity anomalies reported by Palomeras et al.^23^ (Supplementary Material 5). Importantly, our model requires significantly less displacement of the Alboran Domain, located in the upper plate of the subduction system, and implies reduced slab rollback and rotation compared to the scenario illustrated in panel A of Fig. 8. In this configuration, subduction of the oceanic lithosphere beneath the western part of the orogen exerted a pull-down force on the adjacent continental lithosphere, driving its subduction to the east of the Antequera Fault. The climax of this process—recorded in the metamorphic evolution of the Nevado-Filábride Complex (NFC), the lowermost unit of the Internal Zone— likely occurred shortly before the Aquitanian e.g.^86,87^. The subsequent initiation and eastward propagation of the Antequera tear progressively disrupted slab continuity, effectively halting the pull-down and triggering the onset of the NFC exhumation.

These geodynamic models agree that the process of slab rollback influenced the kinematics of the subduction system and facilitated the development of tear faults, segmenting the lithospheric slabs. However, they differ on how slab segmentation occurred. In this paper, the 3D slab geometry reconstruction, along with magmatic products and high-temperature metamorphism, strongly supports that tearing along the ENE-WSW Antequera transfer fault induced asthenospheric upwelling and triggered a rapid thermal pulse, implying that slab segmentation was primarily controlled by the Antequera Fault Zone, rather than the previously proposed E-W tear faults. This finding aligns with kinematic reconstructions, suggesting that the Betic-Rif Internal Zone represents a segment of the Maghrebian margin that collided with the South-Iberian margin after oceanic crust consumption, implying modest northwestward displacements^24,25,28,88^. Within this scenario, the Antequera Fault is defined as a transfer structure that affected both the Maghrebian and South-Iberian margins before orogenesis, and persisted in both the upper and lower plates after subduction and collision. In the upper plate it has a surface expression, while in the lower plate, it represents a vertical tearing. Furthermore, the seismicity distribution and present-day displacement field derived from GPS data e.g.^49^ proves that the Antequera Fault Zone remains as a dextral transfer fault, accommodating strain partitioning between the western and central Betic Cordillera (Fig. 1B). Given this framework, it is concluded that the Gibraltar Arc formed by the westward escape and retreat of the oceanic slab, confined to the south of the Antequera Fault Zone. This process explains the formation of arcuate structures by crustal shortening at the orogenic front, synchronously to the development of the western Alboran Basin by extension in the hinterland. Calc-alkaline magmatism occurred during a late to post-collisional phase, from the middle Miocene to the Pliocene, forming an ENE-WSW trending band along the Alboran Basin. These volcanic rocks intrude the upper plate of the subduction zone, and their spatial distribution reflects the geometry of the subducted oceanic crust (Fig. 8C). The inhibition of melting within the mantle wedge during subduction and collision led to mantle fertilization beneath Alboran, creating a high potential for magma generation during subsequent extensional events in the region.

Our model, proposing a slab length of ~ 100–150 km, shows that localized rollback and associated mantle flow can occur even in small-scale subduction systems. While large-scale rollback is typically linked to longer slabs, several studies have shown that rollback and toroidal mantle flow start from the onset of subduction e.g.,^89,90^, and therefore can also develop with more limited slab lengths, especially when aided by plate convergence forces^91^. This highlights that slab segmentation and retreat in the Betic-Rif system are mechanically feasible despite the modest slab dimensions proposed.

Slab tearing linked to the inversion of former rift-related transfer faults and fracture zones has been documented in various subduction zones worldwide, such as Central America (Tehuantepec Ridge;^92,93^), the Scotia Arc^94^ and the subducted Juan de Fuca plate^95,96^, where inherited structures controlled slab segmentation, mantle flow, and volcanism. Our results add new insights into these topics by linking vertical slab tearing along the Antequera Fault Zone with localized thermal pulses in the Western Betic Cordillera, recorded by zircon growth and leucogranitic magmatism. These findings support a geodynamic model in which slab rollback and tearing were primarily accommodated along the Antequera Fault Zone, critically influencing the tectonic evolution of the Betic-Rif orogen. Overall, the study underscores the key role of asthenospheric upwelling in triggering localized crustal melting and metamorphism, and refine our understanding of how inherited structures influence subduction dynamics and magmatism in complex 3D tectonic settings.

## Methods

This study integrates (a) previously published datasets, which include receiver function images, seismic sections and boreholes from the Guadalquivir Basin, geological cross-sections, and seismicity/focal mechanisms; and (b) new datasets generated herein, including a geological map of the Antequera Fault Zone, an updated map of magmatic occurrences in the Western Betics, new geochemical data for the Málaga dykes, and SHRIMP U–Pb zircon geochronology on leucogranites and migmatites. The methods applied to acquire, process, and interpret these datasets are described in the following sections.

### Construction of a 3D slab interface model

The 3D model of the subducting slab interface integrates seismic reflection profiles, borehole data, and both published^24^ and unpublished cross-sections, constraining the top of the Iberian basement to depths of up to ~ 9 km. Additionally, published P-to-S converted receiver function migrated images, when combined with the distribution of seismicity, allow for the imaging of deeper lithospheric structures. Key seismic reflection lines analyzed in this study include: AES: 84–21; BT: 8A, 8B; MA: 3; RG: 1–2, 3–2, 4–2, 11, 21, 87_02; RGKO-89: 01; RGKO-91: 03, 10, 10–22, 12, 18, 20; RS: 6, 8, 14, 20; S82: 32A, 34; S82A: 2, 5, 8, 9, 11, 16, 37, 141, 142, 143; S83: 40, 48, A-221, A-222; S84: 40; and 84-G: 14. Boreholes critical to the structural interpretation, either by penetrating the basement or providing minimum depth constraints to its top, include: Almonte-1; Asperillo-1; Bética 18–1; Carmona-3, -4, -5, -6; Casa Nieves-1; Córdoba A-2; Córdoba B-2; Écija-2; Fuensanta-1; Huelva-1; Isla Mayor-1; Moguer-1; Murcia B-1; Nueva Carteya-1; Río Guadalquivir H-1; Río Guadalquivir N-1; Río Segura G-1; Socovos-2. Time-to-depth conversion of the seismic lines was performed using stacking velocity models derived during seismic processing, and where applicable, supplemented by interval velocity data from adjacent boreholes. All seismic and borehole data are archived and accessible through the Spanish Hydrocarbon Database (http://info.igme.es/infogeof/).

Receiver function imaging is highly sensitive to seismic velocity contrasts, making it particularly effective for delineating major lithospheric discontinuities such as the Moho, subducting interfaces, and potential tear faults. Therefore, the receiver functions and seismicity data up to 2024^97^, can serve to constrain the deep geometry of the subducting slab interface beneath the Internal Zones of the Betic Cordillera. The top and base of the subduction slab interface was identified using the available receiver-side forward- and back-scattered mode conversions^54–57^. Therefore, the receiver functions and seismicity distribution (up to 2024^97^), can serve to constrain the deep geometry of the subduction slab interface beneath the Betic Cordillera. Notably, abrupt terminations in the receiver function interfaces and deep seismicity were used to identify lateral slab tear boundaries. The slab geometry has been constrained down to a depth of 130 km. Below this depth, if present, the slab has not been examined due to lack of definition in the receiver function models and absence of seismicity. Earthquakes located within 5 km to receiver function profiles were projected perpendicularly onto the P-to-S converted images in order to enable an integrated interpretation of seismicity distribution and lithospheric structure. The interpreted boundaries were interpolated by Delaunay triangulation using MOVE software (Petex Ltd.). The resulting 3D surface was then smoothed to remove small-scale anomalies caused by irregular data distribution, using a spatial filter threshold of 5 km.

### Location and geochemistry of magmatic suites

The distribution of magmatic products, including the location of the Málaga and the leucogranite dykes, were investigated by reviewing existing geological maps^98^ and references therein] and conducting new field mapping of the Western Betic Cordillera^99–101^ (Supplementary Material 1 and 2). To delve into the geochemistry of the Málaga dykes , published results^47,63^, together with two new samples collected specifically for this study, were utilised. Trace element contents were plotted in multiple geochemical diagrams to categorize the basaltic magmatism linked with the supra-subduction system (Fig. 5). To avoid issues related to element mobility arising from alteration and/or hydrothermal processes when investigating the nature of the source area for these basalts and their petrogenesis, a suite of geochemical diagrams were employed. These diagrams focus on incompatible trace elements, including Th, Nb, and Yb, among others, which are known to remain relatively immobile during secondary processes. Additionally, the MORB-normalized Th *vs.* Nb diagram, as well as the Th/Yb *vs.* Nb/Yb and TiO_2_/Yb *vs.* Nb/Yb plots serve as a valuable tectonic discrimination tool, as proven with numerous geochemical diagrams or representative samples from similar geodynamic contexts^102^.

### Zircon geochronology

Four samples of leucogranite and three of migmatites were used for the geochronological analyses (Supplementary Material 1, 3 and 4). Zircon separation was accomplished by traditional techniques using dense liquids and magnetic separation (Frantz) at the University of Huelva (Spain). Crystals free of impurities and fractures were selected by hand-picking under a binocular microscope. Analyses were carried out in the SHRIMP II microprobe at the Shrimp Ion-Microprobe Laboratory (IBERSIMS) of the University of Granada (Spain). Zircon concentrates were cast on a 3.5 cm diameter epoxy mount, together with zircon standards (TEMORA, SL13 and GAL zircon) and documented by a Zeiss EVO 150 Scanning Electron Microscopes (SEM) equipped with a cathodoluminescence detector (SEM-CL). Mounts were coated with gold (80-nm thick) and inserted into the SHRIMP for analysis. Each selected spot was rastered with the primary beam during 120 s prior to analysis and then analyzed over 6 scans following the isotope peak sequence: ^196^Zr_2_O, ^204^Pb, 204.1 background, ^206^Pb, ^207^Pb, ^208^Pb, ^238^U, ^248^ThO, ^254^UO. Every peak of each scan was measured sequentially 10 times with the following total counts per scan: 2 s for mass 196; 5 s for masses 238, 248, and 254; 15 s for masses 204, 206, and 208; and 20 s for mass 207. The primary beam, composed of ^16^O^16^O^2+^, was set to an intensity of 4 to 5nA, using a 120-nm Kohler aperture, which generates 17 × 20-μm elliptical spots on the target. The secondary beam exit slit was fixed at 80 nm, reaching a resolution of about 5000 at 1% peak height. Mass calibration was carried out on the GAL zircon (ca. 480 Ma, very high U, Th and common lead content; Montero et al.,^103^. Sessions initially involved the measurement of SL13 zircon^104^, used as a concentration standard (238 ppm U]. TEMORA zircon (ca. 417 Ma,^105^), used as isotope ratio standard, was measured every 4 unknowns. The result for each isotope was calculated as the value at the mid-time of the analysis resulting from the regression line. ^206^Pb/^238^U was estimated from the measured ^206^Pb^+^/^238^U^+^ and UO^+^/U^+^, following the method of Williams^106^. Plotted and tabulated analytical uncertainties are 1σ precision estimates. Uncertainties are 95% confidence limits (tσ, where t is the student’s t multiplier) and include the uncertainty in the Pb/U calibration (ca. 0.3–0.5%). Ages were calculated using the constants recommended by the International Union of Geological Sciences (IUGS) Subcommission on Geochronology^107^.

## Supplementary Information

Supplementary Information 1.

Supplementary Information 2.

Supplementary Information 3.

Supplementary Information 4.

Supplementary Information 5.

## Data Availability

All geological, geochemical and geochronological data generated and analysed during this study are included in this published article [and its supplementary information files.
