# Supplementary material for: Evidence of slab tearing on an inherited Mesozoic rift transfer fault in the Betic Cordillera
Source: Sci Rep. 2025 Aug 7;15:28855. doi: 10.1038/s41598-025-13168-z (PMC12331968; doi:10.1038/s41598-025-13168-z)
Supplement: Supplementary file 3 — Supplementary Information 3. [file 41598_2025_13168_MOESM3_ESM.docx]

**Supplementary Material 3: Description of samples and zircon populations.**

Leucogranite samples were collected from areas within ultramafic massifs that have been significantly affected by brittle deformation and serpentinization, predominantly near major structural lineaments reactivated during the Miocene contractional episode.

Sample Carra-02 represents a small intrusive body of Crd-leucogranites, approximately 100 meters in extent, located to the northeast of the study area. The Crd, heavily pinnitized, imparts a greenish-white color to the outcrop. The leucogranite exhibits a ductile-brittle fabric with a roughly N-S orientation, continuous with other leucogranite dikes and serpentinized zones emplaced within the peridotites of this sector.

The zircon sample from Carra-02 leucogranites comprises several simple crystals, with variable sizes ranging from 300 microns in length to small fragments of acicular zircons, and euhedral to subhedral prismatic zircons composed of an inherited core and an outer concentric growth. The cores yield inherited ages from the metasedimentary protolith, spanning from the Lower Carboniferous to the Paleoproterozoic. A few Permo-Triassic ages, representing metamorphism associated with the first extensional (Díaz-Alvarado et al., 2024), are present in some rims and internal zones of complex crystals. Most of the overgrowths and simple zircons give Early Miocene ages, ranging from 26 to 17 Ma. Considering only those results with lower discordance, the most probable age for the emplacement and crystallization of the leucogranitic body is 20.33 ± 0.51 Ma (N=8). These Miocene-aged analyses exhibit Th/U ratios between 0.03 and 0.26, typical of metamorphic- and felsic melt-related growths.

Sample Leuco-05 represents a dense network of leuco- and mesocratic granite dikes and small sub-circular bodies (no more than 50 m²) emplaced to the south of the Sierra Alpujata ultramafic massif, south of the locality of Ojén. These are associated with E-W to ENE-WSW brittle structures that also induce significant serpentinization of the peridotites.

Zircon population from sample Leuco-05 includes small crystals with moderate elongation, ranging from 100 to 150 microns in length, mostly exhibiting an inherited core and variably developed outer growths. Only a few simple acicular zircons are present. The cores of these zircons show inherited ages from the metasedimentary protolith, though the number of data points is insufficient for a significant evaluation (ranging from the Devonian to the Paleoproterozoic). Three highly discordant Mesozoic ages were found among the zircon growths, but most analyses of these external areas yielded Early Miocene ages, with a narrow variation range between 22 and 19 Ma, and a mean age for the most probable emplacement and crystallization of the leucogranite of 20.23 ± 0.32 Ma (N=19). Th/U ratios, ranging from 0.25 to 0.01, indicate crystallization from a felsic melt and metamorphic growths.

Sample SN-63 was collected from a 1-meter thick leucosome rooted in a pelitic metatexite belonging to a small body of metasediments pinched at the tectonic contact between peridotites and Triassic carbonate rocks to the north of the study area.

Morphologically, the zircon population of sample SN-63 is characterized by moderately sized grains (200-250 microns) and simple euhedral prisms. They are marked by low luminescence in cathodoluminescence images and generally exhibit weak zoning. Only two of the few inherited cores were analyzed, yielding one age typical of the metasedimentary protolith (Ediacaran) and another from the Permian metamorphic process. Twenty-six analyses of simple zircons or growths on inherited cores resulted in ages ranging from 22 to 19 Ma, with very low discordance, providing a mean age of 20.52 ± 0.16 Ma. Th/U ratios below 0.15 reflect the high uranium content of these zircons, related to metamorphic processes.

In addition to the results obtained from leucogranites of the study area, we have included the results from zircons in a band of marbles intercalated with nebulites (heterogeneous granites) atop of the peridotites in the Cerro del Robledar area, near the contact between peridotites and carbonates to the north of the Guadaiza unit.

The marbles MA-02 contains few zircons, some of which are complex with inherited cores and concentric growths, while others are simple, small, anhedral, or with a spicular habit. Only two inherited cores could be dated, yielding results similar to those of the samples presented above. The simple crystals and outer growths yielded results ranging from the Eocene to the Early Miocene, with the oldest being discarded due to high discordance (>88%). Results with discordances less than 6% range from 24 to 20 Ma, with a mean age of 20.86 ± 0.64 Ma (N=8). The Th/U ratios of the Early Miocene ages are below 0.1, indicating a metamorphic affinity.

In addition to the leucogranites presented above, three samples of migmatites collected from structural lineaments reactivated during the Miocene have been included in the study. These include a Grt-bearing metatexite to the south of the study area (MA-05) and two mylonitized nebulites (heterogeneous granites) in the N-S structure that connects the northern and southern tectonic contacts of the peridotites of the Guadaiza unit. These samples have been described in detail in Díaz-Alvarado et al. (2024), and their main geochronological results are included in this study. This work shows that only those metamorphic and migmatitic units near these reactivated lineaments exhibit a well-defined Miocene cluster, whereas in other samples from different locations, the thermal effect of the Miocene process disturbs previously established isotopic ratios, generating a scatter of rejuvenated ages without consolidating a group of ages as observed in the migmatites selected for this study.
