# Supplementary material for: Evidence of slab tearing on an inherited Mesozoic rift transfer fault in the Betic Cordillera
Source: Sci Rep. 2025 Aug 7;15:28855. doi: 10.1038/s41598-025-13168-z (PMC12331968; doi:10.1038/s41598-025-13168-z)
Supplement: Supplementary file 5 — Supplementary Information 5. [file 41598_2025_13168_MOESM5_ESM.pdf]

Supplementary Material 5

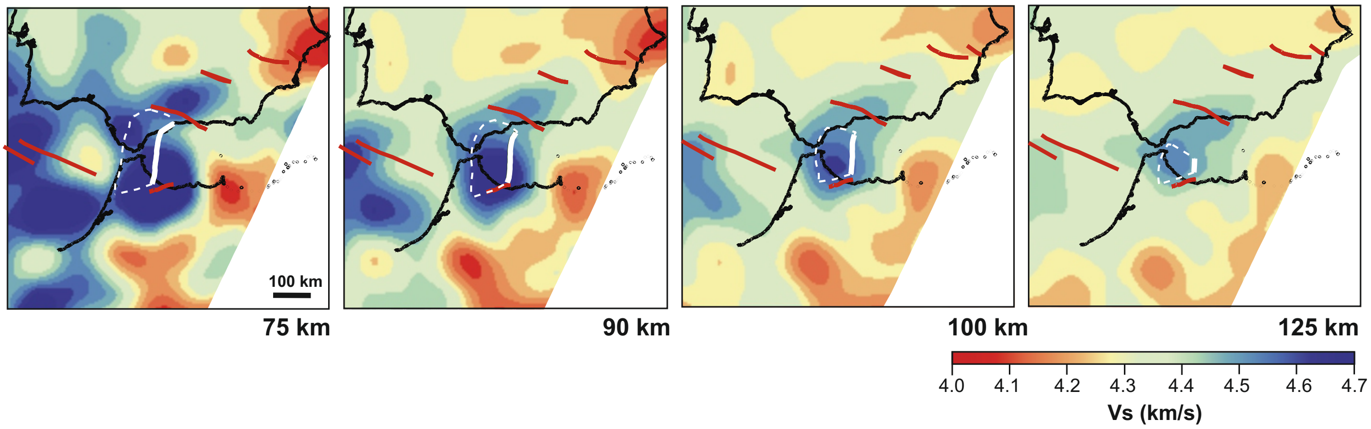

S5. Maps of absolute shear-wave velocity at various depths (Palomeras et al., 2017; courtesy of Inmaculada Palomeras), showing the spatial correlation with our subducting slab model (solid white line). A lithospheric thickness of 100 km and the calculated slab dip were used to delineate the base of the subducting lithosphere (dashed white line).Maps generated with Move Suite 2017.1 (https://www.mve.com/) and Corel Draw Graphics Suite X8 (https://www.coreldraw.com/).
